# The Relationship Between Sedentary Behavior, Back Pain, and Psychosocial Correlates Among University Employees

**DOI:** 10.3389/fpubh.2019.00080

**Published:** 2019-04-09

**Authors:** Fahad Hanna, Rua N. Daas, Tasneem J. El-Shareif, Haneen H. Al-Marridi, Zaina M. Al-Rojoub, Oyelola A. Adegboye

**Affiliations:** ^1^Master of Public Health Program, School of Public Health, Torrens University, Melbourne, VIC, Australia; ^2^Department of Public Health, College of Health Sciences, Qatar University, Doha, Qatar; ^3^Department for Management of Science and Technology Development, Ton Duc Thang University, Ho Chi Minh City, Vietnam; ^4^Faculty of Mathematics and Statistics, Ton Duc Thang University, Ho Chi Minh City, Vietnam

**Keywords:** back pain, physical activities, wellness, sitting too much, occupational hazard

## Abstract

**Objectives:** This study aims to investigate the relationship between levels of sedentary behavior, physical activity, and back pain and their psychosocial correlates among university employees.

**Methods:** A cross-sectional study was conducted on both academic and non-academic professional staff at Qatar University. The data collection instrument was a combination of the International Physical Activity Questionnaire (IPAQ), the Global Physical Activity Questionnaire (GPAQ) and the Acute Low Back Pain Screening Questionnaire (ALBPSQ).

**Results:** A total of 479 individuals (57% females) participated in the cross-sectional study. Two hundred and ninety three (61.2%) reported to have experienced back pain. The covariates adjusted odds ratios (aORs) showed that vigorous physical activity was a protective variable for those who experienced lower back pain [aOR = 0.84, 95%CI (0.56–0.98)], both lower and upper back pain [aOR = 0.61, 95% CI (0.33–0.87)] and either lower or upper back pain [aOR = 0.76, 95%CI (0.51–0.85)], respectively. Back pain was significantly higher in females than males (aORs: 1.37–2.21). Similarly, sedentary behavior (too much sitting) was significantly associated with those who experienced either LBP or UBP [aOR = 1.74, 95% CI (1.19, 2.57)]. All back pain categories were found to be significantly associated with those who reported a depressed mood.

**Conclusions:** These findings suggest that sedentary employees are exposed to increasing occupational hazards such as back pain and mental health issues. Strategies should aim to reduce sitting time with planned and feasible physical activity interventions to be incorporated into the workplace health promotion policy to help prevent back pain, back injuries, and mental health complications.

## Introduction

Modern day research concerning sedentary behavior and physical inactivity has shown a rise in musculoskeletal pain which has increasingly become more prevalent in the last 40 years (1). Sedentary behavior is defined as a distinct class of behaviors (e.g., Sitting) that is characterized by little physical movement and activities that involve low energy expenditure of < 1.5 metabolic equivalent units (2–5). Dramatic decreases in movement and activity are not only resulting in the development of various chronic diseases such as cardiovascular diseases (6, 7) and obesity (8), but may also leads to the rise of musculoskeletal disorders (MSDs), including pain and disability (9).

Back pain (BP) is one of the most debilitating conditions inflicting grief, discomfort, and disability to its bearer. It affects the person's mental well-being as well as his/her efficiency in performing day-to-day tasks, hence, affecting productivity and consequently weakening the economy (10–12). As we are moving to a highly-industrialized and highly robotic era where sedentary work is very common, it is rather essential to tackle such health consequences systematically. Musculoskeletal Disorders (MSDs), especially in the neck, shoulders, and lower back regions are common among office workers worldwide, due to the absence of physical activity, lack of movement as well as the adopted sedentary behavior (13). The burden of back pain cannot be overemphasized. For example, it has been reported that low back pain (LBP) is the second cause of disability in the United States, with more than 80% of people experiencing LBP at one point in their lives (14–16). LBP was also found to be more prevalent in females than males; additionally, people with a higher body mass index (>26.0 kg/m^2^) are also more likely to suffer from it (17).

The most likely population to be prone to developing MSDs and to be affected by them, are those who spend most of their working hours sitting, commonly due to the nature of their work (10). For instance, it has been established that sitting time is proportionately associated with LBP among blue-collar workers in Sweden (18). On the other hand, in a study that introduced an activity-based work office environment, participants were twice as likely to report LBP at baseline (the inactive baseline environment) than after introducing an activity-based office (19).

Lack of vigorous physical activities may lead to LBP (20) and may have a negative impact on job satisfaction (21). Prolonged sitting or standing, static posture and uncomfortable back support were all found to be associated with LBP, shoulder pain and upper back pain (UBP) (13, 22). For instance, a study of bank office workers in Kuwait indicates that pain and disability in the neck, lower back, shoulders, and upper back are associated with longer job durations, and being older (23). On the other hand, MSDs are known to be associated with the prevalence of other conditions such as psychological disorders. A study in Qatar found that episodes of LBP were associated with psychological distresses such as somatization, depression, and anxiety in those who attended primary health care centres (24).

In the Arabs region, particularly in the Gulf, additional elements such as harsh weather, organizational structure, traditional roles for women, lack of social support, and use of housemaids serve as barriers for physical activity and have further exacerbated musculoskeletal and other chronic diseases (25, 26). Furthermore, according to the study of Global Burden of disease in 2010, LBP was found to be one of the top three causes of disability-adjusted life years (DALYs) in Qatar (27).

Physical activity is the most basic, simple and well-known preventive and therapeutic measure for MSDs such as back pain, particularly when the problem is caused by sedentary behavior (18, 28, 29). Therefore, the application of some of the work-based activities and interventions may be beneficial in our increasingly sedentary behavior. The academic sector, and particularly higher education, where academics invest lengthy hours in teaching preparation and research, may provide the right environment to address and raise awareness of the risks of prolonged sitting and lack of activity and their potential implication on the health of these individuals. The main goal of this study was to highlight the risks of leading a sedentary behavior and to provide an incentive for the design and implementation of simple and brief interventions to minimize the potential damage. The objective was to investigate the relationship between levels of sedentary behavior, physical activity, and back pain and their psychosocial correlates among university employees.

## Materials and Methods

### Study Design and Participants

Five hundred and fifty-four (19%) participants among a pool of 2,906 academic and non-academic employees (professional staff) at Qatar University were recruited for this cross-sectional study using non-random sampling (convenience sample). The inclusion criteria encompassed full time academic and non-academic employees of Qatar University. Pregnant women were excluded. Out of 554 participants who agreed to participate, 74 (13.4%) were unavailable or refused to participate at the time of the data collection. The final number of participants who completed the questionnaires and were included in the analysis was 479 employees. This final number consisted of 240 academic staff and 239 professional staff aged 25 years and older. Each participant was asked to complete a combined validated questionnaire on health and well-being including information regarding sitting time as a measure of sedentary behavior, physical activity, back pain, and well-being.

### Instruments

For this study, a combination of three questionnaires, the Global Physical Activity Questionnaire (GPAQ), the International Physical Activity Questionnaire (IPAQ), and the Acute Low Back Pain Screening Questionnaire (ALBPSQ) were used. A new 24-item questionnaire was formulated from these three questionnaires. Table S1 presents the full list of items in the questionnaire.

The questionnaire used in the study consisted of four sections:

**Section 1:** Participants baseline characteristics such as gender, age, and profession (academic/non-academic).

**Section 2:** This section was based on the Global Physical Activity Questionnaire developed by the World Health Organization (WHO) (30) The questionnaire was tested by the WHO research program where its reliability and validity were confirmed. In addition to that, the questionnaire was used in more than 100 countries, which further supports its effectiveness and validity across different cultures and settings (30). Measurements such as duration of sitting during and outside working hours (e.g., while traveling by a car or bus) as well as time spent sitting at home was used a measure for sedentary behavior. Following Owen et al. (6), we classified levels of sitting time as sedentary behavior (Too much sitting, hereafter) if the duration of sitting is more than 10 h.

**Section 3:** The questionnaire used in this section was the International Physical Activity Questionnaire (IPAQ) (31). Its reliability and validity has been previously tested across 12 countries; and it has been proven to be a successful questionnaire for quantifying physical activity across many international population-based studies (31). The levels of physical activity of the participant were measured and as light, moderate or vigorous intensity exercise. These levels were assessed according to the time spent exercising per day in a typical week as reported by the participant.

**Section 4:** This section was based on the Acute Low Back Pain Screening Questionnaire (ALBPSQ) (32). The questionnaire was endorsed by guidelines from New Zealand to recognize those who are at risk of developing non-specific LBP (33). The levels of back pain were measured using duration and intensity of the pain. The perceived beliefs regarding the psychological impact of pain on the participant was also assessed using a Likert rating scale from 0 (no pain) to 5 (pain as bad as it could be).

### Reliability and Validity of Instruments

The GPAQ, IPAQ, and ALBPSQ questionnaires, including implementation, reliability, and validity for several countries, have been discussed in detail elsewhere (30–32). The psychometric properties of GPAQ, IPAQ, and ALBPSQ questionnaires were investigated for the population in this study. The reliability and validity of our instruments for the Qatari population was in agreement with those previous studies (30–32). Spearman's scores for validity for self-reported questionnaires (combined) were 0.76 and 0.85 for reliability.

### Statistical Analysis

Data analysis in this study was performed using IBM SPSS Statistics for Windows, Version 23. Descriptive analyses were presented as means and standard deviations for continuous variables, and frequencies and percentages for categorical variables. The chi-square test was used to compare participant's baseline categorical variables. Logistic regression was applied to the categorical response variables to investigate the relationship between back pain and variables of interest (sitting time, physical activity, and stress), gender, age, and profession. Odds ratios (OR) and 95% confidence interval were presented for categorical variables. Inference was based on 5% level of significance.

## Results

The baseline demographic characteristics of participants are listed in Table 1. Of the 479 participants (57% females) in this study, more than half (293, 61.2%) reported that they had experienced LBP or UBP (38% males vs. 62% females, *p* = 0.008). Sixty three participants (13.2%) reported to have both LBP and UBP, 181 (37.9%) reported to have only LBP and 46 (9.6%) reported to have only UBP, while 187 (39.2%) reported no BP at all. The prevalence of back pain (either LBP or UBP or both) was higher among the age group 31–40 years. There were more non-academics who had experienced either LBP or UBP than academics (33.2% vs. 28.0%, respectively, *p* = 0.022). Twenty-six percent of people doing vigorous physical activity had either LBP or UBP in comparison with 35% of people with no vigorous physical activity (*p* = 0.034) (Table 1). The incidence of LBP was significantly associated with moderate physical activity and being depressed. There was no significant association between the baseline characteristics and UBP, although being depressed reached borderline significance (*P* = 0.065).

**Table 1:** Baseline characteristics of the study population with any back pain (upper or lower) and those with both lower and upper back pain.

**Risk factor**	Lower *N* = 181 (37.8%)^*^	***P***	Upper *N* = 46 (9.6%)^*^	***P***	Upper or lower *N* = 293 (61.2%)^*^	***P***	Both back pain *N* = 63 (13.2%)^*^	***P***
**GENDER**								
Male	77 (16.1%)	0.881	18 (3.8%)	0.579	112 (23.4%)	0.008	16 (3.40%)	0.002
Female	104 (21.8%)		28 (5.9%)		181 (37.8%)		47 (9.90%)	
**AGE**								
21-30	47 (9.9%)	0.859	15 (3.2%)	0.448	78 (16.3%)	0.991	14 (2.90%)	0.438
31-40	83 (17.4%)		19 (4.0%)		133 (27.8%)		30 (6.30%)	
above 40	51 (10.8%)		12 (2.5%)		82 (17.1%)		19 (4.00%)	
**PROFESSION**								
Non-academic	91 (19.1%)	0.953	27 (5.7%)	0.228	159 (33.2%)	0.022	39 (8.20%)	0.044
Academic	90 (18.9%)		19 (4.0%)		134 (28.0%)		24 (5.00%)	
**VIGOROUS PA**								
No	100 (21.1%)	0.477	21 (4.4%)	0.263	168 (35.2%)	0.034	44 (9.30%)	0.005
Yes	80 (16.8%)		25 (5.3%)		124 (26.0%)		19 (4.00%)	
**MODERATE PA**								
No	72 (15.4%)	0.020	17 (3.6%)	0.611	126 (26.9%)	0.200	34 (7.30%)	0.028
Yes	104 (22.3%)		28 (6.0%)		161 (34.3%)		29 (6.20%)	
**TOO MUCH SITTING**^**a**^								
Yes	105 (23.1%)	0.194	28 (6.1%)	0.681	177 (38.7%)	0.002	41 (8.9%)	0.097
No	67 (14.6%)		16 (3.5%)		104 (22.7%)		20 (4.4%)	
**DEPRESSED MOOD**^**b**^								
Yes	30 (10.5%)	< 0.001	16 (5.6%)	0.065	71 (24.9%)	0.085	25 (8.8%)	0.003
No	147 (51.6%)		28 (9.8%)		213 (74.7%)		38 (13.3%)	

* *Percentage out of the total number in each category*.

a *Participants were asked how much time do you usually spend sitting or reclining on a typical day? Too much sitting was categorized as sitting for more than 10 h*.

b Depressed mood was initially categorized as “not at all: 0” to “extremely depressed 5” but was dichotomized as “No” vs. “Yes.”

Tables 2–**5** presents the results on estimated odds ratio (OR) with their 95% confidence interval (CI) from the covariates adjusted logistic regression models. The associations between experiencing back pain (LBP, UBP, both and either) and physical activity are presented in Table 2. There was a significant association between back pain (LBP, both LBP and UBP, and either LBP or UBP) and vigorous physical activity with those reporting vigorous physical activity being less likely to experience LBP [OR = 0.84, 95%CI (0.56–0.98)], both back pain [OR = 0.61, 95% CI (0.33–0.87)] and either LBP or UBP [OR = 0.76, 95%CI (0.51–0.85)], respectively. Interestingly, moderate physical activity was found not to be associated with having upper and lower back pain separately; [OR = 1.02, 95%CI (0.51–2.05)] and [OR = 1.11, 95%CI (0.73–1.68)], respectively. The incidence of all categories of back pain were significantly increased for females. The estimated odds ratios of experiencing back pain for females were 1.57 for UBP, 1.37 for LBP, 2.21 for both types of back pain and 1.96 for either back pain.

**Table 2:** Relationship between back pain and levels of physical activity.

**Risk factor**	**Upper back pain**	**Lower back pain**	**Both back pain**	**Upper or lower**
	**OR (95% CI)**	**OR (95% CI)**	**OR (95% CI)**	**OR (95% CI)**
Female gender	**1.57 (1.14–2.27)**	**1.37 (1.01–1.53)**	**2.21 (1.14–4.27)**	**1.96 (1.45–2.21)**
Age (ref 21–30)				
31–40	0.82 (0.39–1.70)	1.13 (0.72–1.80)	1.56 (0.77–3.12)	1.23 (0.77–1.96)
Above 40	0.89 (0.36–2.13)	0.94 (0.55–1.63)	1.89 (0.84–4.25)	1.11 (0.65–1.90)
Profession: academics	0.70 (0.36–1.37)	1.04 (0.69–1.55)	0.66 90.36–1.21)	0.74 (0.50–1.11)
Vigorous PA	0.88 (0.75–2.94)	**0.84 (0.56–0.98)**	**0.61 (0.33–0.87)**	**0.76 (0.51–0.95)**
Moderate PA	1.02 (0.51–2.05)	1.11 (0.73–1.68)	0.77 (0.43–1.38)	0.91 (0.60–1.38)

*Bold values represent significant at 0.05*.

Table 3 shows the associations between depressed mood and different categories of back pain. The covariates adjusted model showed that participants who reported they had experienced depressed mood are more likely to have UBP [OR = 1.96, 9 5%CI (0.96–3.97)], LBP [OR = 1.33, 95%CI (1.19–1.58)], both LBP and UBP [OR = 2.33, 95%CI (1.25–4.34)], either LBP or UBP [OR = 2.28, 95%CI (1.22–2.56)].

**Table 3:** Relationship between depressed mood and back pain after adjusting for age and gender.

**Risk factor**	**Upper back pain**	**Lower back pain**	**Both back pain**	**Upper or lower**
	**OR (95% CI)**	**OR (95% CI)**	**OR (95% CI)**	**OR (95% CI)**
Female gender	0.74 (0.36–1.57)	0.74 (0.41–1.31)	0.51 (0.25–1.02)	1.59 (0.13–3.57)
Age (ref 21–30 years)				
31–40 years	0.78 (0.36–1.69)	0.87 (0.47–1.61)	1.61)0.77–3.35)	3.85 (0.28–5.63)
Above 40 years	0.71 (0.28–1.81)	0.69 (0.34–1.43)	2.33 (0.99–3.52)	1.68 (0.99–2.45)
Profession: academics	0.85 (0.42–1.72)	1.52 (0.88–2.60)	0.67 (0.36–1.27)	0.15 (0.16–1.44)
Depressed mood^a^	**1.96 (0.96–3.97)**	**1.33 (1.19–1.58)**	**2.33 (1.25–4.34)**	**2.28 (1.22–2.56)**

a *Depressed mood was initially categorized as “not at all: 0” to “extremely depressed 5” but was dichotomized as “No” vs. “Yes.” Bold values represent significant at 0.05*.

In the covariates adjusted model shown in Table 4, there was no significant association between sitting too much and back pain (UBP, LBP, and both back pain). However, participants who reported sitting too much are more likely to experience either lower or upper back pain [OR = 1.74, 95% CI (1.19–2.57)]. Female gender are significantly more likely to experience both back pain [OR = 2.67, 95%CI (1.38–5.17), and either UBP or LBP (OR = 1.55, 95%CI (1.03–2.37)] than their male counterpart.

**Table 4:** Association between back pain and too much sitting after adjusting for age, gender, and profession.

**Risk factor**	**Upper back pain**	**Lower back pain**	**Both back pain**	**Upper or lower**
	**OR (95% CI)**	**OR (95% CI)**	**OR (95% CI)**	**OR (95% CI)**
Female gender	0.97 (0.49–1.92)	1.02 (0.67–1.54)	**2.67 (1.38–5.17)**	**1.55 (1.03–2.37)**
Age (ref 21–30 years)				
31–40 years	0.79 (0.38–1.69)	1.13 (0.71–1.82)	1.69 (0.84–3.38)	1.29 (0.80–2.09)
Above 40 years	0.86 (0.35–2.07)	0.97 {0.56–1.70)	1.95 (0.86–4.42)	1.19 (0.68–2.06)
Profession: academics	0.75 (0.38–1.49)	1.08 (0.71–1.64)	0.73 (0.40–1.32)	0.83 (0.55–1.26)
Too much sitting^a^	1.26 (0.65–2.41)	1.29 (0.87–1.91)	1.61 (0.89–2.89)	**1.74 (1.19–2.57)**

a *Participants were asked how much time do you usually spend sitting or reclining on a typical day? Too much sitting was categorized as sitting for more than 10 h. Bold values represent significant at 0.05*.

In Table 5, the relationship between depressed mood and sitting too much was explored. After adjusting for age, gender and profession, there was a trend toward significant association between depressed mood and sitting too much [OR = 1.41, 95% CI (0.77–2.63), *P* = 0.056]. Other significant risk factors exist with participants aged 40 years and above [OR = 0.35, 95% CI (0.16–0.73)], and academic staff [OR = 0.54, 95% CI (0.31–0.92)].

**Table 5:** Association between sitting too much and depressed mood after adjusting for age and gender.

**Risk factor**	**OR (95% CI)**	***P***
Female gender	0.61 (0.34–1.09)	0.106
Age (ref 21–30 years)		
31–40 years	0.58 (0.30–1.11)	0.221
Above 40 years	**0.35 (0.16–0.73)**	**0.012**
Profession: academics	**0.54 (0.31–0.92)**	**0.021**
Depressed mood^a^	1.41 (0.77–2.63)	0.056

a *Depressed mood was dichotomized as “No” vs. “Yes.” Bold values represent significant at 0.05*.

## Discussion

This cross-sectional study aimed to investigate the relationship between sedentary behavior, particularly sitting too much, physical activity and back pain in Qatar University employees. It also aimed to explore the impact of both sitting too much and back pain on the well-being of these employees. Our study found that the most prevalent type of back pain amongst Qatar University employees is LBP. The results also revealed higher prevalence of any back pain among University professionals compared with academics.

The incidence of LBP among university employees in this study was 37.8% (61.2% experienced back pain). Our findings are consistent with those reported from other studies conducted on office-based workers in the region. A previous Iranian study, based on university office workers reported a prevalence of LBP of 58.2% (21). Another study conducted on bank office workers in Kuwait, stated that prevalence of LBP was 51.1% of its total participants (23).

We found vigorous physical activity to be strongly but inversely associated with LBP, both LBP and LBP and either LBP or UBP. With regard to risk factors in the adjusted models, we did not find a clear association between back pain and age, and profession (academic vs. non-academic). Females were still more likely to report back pain than males in this population, perhaps because more of the non-academic staff were females. These results are consistent with previous studies, which concluded that females were more likely to have back pain (33). Similarly, Bawab et al. (17) found that females were more likely to suffer from LBP than males. These findings are in agreement with an earlier study that revealed that being a female is a risk factor for having MSDs (22).

When studying the association between different categories of back pain with physical activity, the covariate adjusted models showed a strong negative association between back pain and vigorous physical activity indicating a protective factor. In other words, those with back pain were less likely to report vigorous physical activity. The effect of physical activity has been shown to be beneficial in previous studies (23). These findings were congruent with a Kuwait bank office study, which showed a significant correlation between LBP and low levels of physical activity (23), and with a recent Thai study that investigated the effectiveness of activity based work environments on LBP (20). Similarly, an Australian study concluded that LBP was lower in the activity based-work environment than the inactive baseline environment, in that participants were twice more likely to report LBP at baseline compared with during the intervention (19). In addition, exercise programs consisting of muscle stretching and endurance training is an effective intervention to reduce the incidence of LBP in otherwise healthy office workers (19).

Our study also showed that employees who sit too much were more likely to experience either lower or upper back pain. Our finding agrees with other studies; office workers involved in prolonged sitting during their work shift were more likely to report LBP (20, 29). Other established risk factors for reporting LBP were frequent computer use by (92%) and sitting for more than 2 h/day (88%) during a work day (34–36).

With respect to psychological aspects of our investigation, it was observed that those who experienced any form of back pain were more likely to show a depressed mood after adjusting for other covariates. Lower back pain has been associated with a higher level of disability in comparison with UBP. The association between upper back pain and depressed mood in our study is consistent with findings from previous studies that reported a causal relationship between upper back pain and depression symptoms in adults (34, 36, 37).

## Conclusions and Recommendations

The cross-sectional analysis revealed a significant association between both upper and lower back pain and vigorous physical activity with the latter being protective of back pain in sedentary type workers. The findings of this research revealed that those who have back pain are more likely to experience depression. Sitting too much was also found to be associated with back pain. This study suggests that sedentary type office workers, who form a significant proportion of all workers, are in vital need of workplace health promotion. These findings are of particular importance for Qatar and the Gulf Cooperation Countries and similar geo-climate regions since environmental conditions such as harsh weather do not permit outdoor activities and outdoor exercise for the majority of the year, to compensate for the inactivity caused by sedentary behavior. In such circumstances, where activity is limited, vigorous physical activity and workplace health promotion and interventions should be on a priority list for policy makers, institutional regulations, and occupational health and safety managers. Health promotion and interventions should not only aim at increasing the awareness of modern-day occupational hazards and the risk associated with prestigious and privileged jobs that require no physical efforts or exertion, but should go beyond that to provide simple, brief, and applicable interventions. Jobs such as those in academia that require and demand “too much sitting” and prolonged working hours should be targeted for proportionate interventional activities to protect against back pain and the psychological traumas associated with both being sedentary and developing musculoskeletal pain. Any proposed interventions should, at first, aim at reducing sitting times and providing alternatives such as task variation to reduce prolonged sitting. Interventions should also aim at introducing systematic and organized moderate intensity activity during working hours as well as the recommendation of regular vigorous physical activity during or outside of working hours. Such interventions will also simultaneously help protect against the increase of chronic disease epidemics such as obesity, diabetes and cardiovascular disease that are also highly prevalent in these environments. Indeed, increasing sitting and sedentary time will exacerbate these conditions and any interventions that aim at limiting sedentary time will potentially protect against the rise of modern day chronic diseases, including mental health.

### Limitations

Our study has a number of limitations. Firstly, the cross-sectional design meant that establishing causation between outcome variables and risk factors was not possible. Secondly, self-reported data gathered by questionnaires are always prone to certain types of biases, such as recall bias and/or responder bias; however, the questionnaires used are internationally recognized and validated and are designed to minimize these types of error and biases. Thirdly, some of the information collected was subjective as no instruments were used in our study to measure pain, sitting time, and other variables of interest. A fourth limitation is that we did not have data on genetic predisposition or the body mass index (BMI) of our participants, which may contribute to back pain. Lastly, responses such as “depressed mood” were self-reported and had a subjective nature. However, the instruments used in this study were extensively tested and validated, despite the self-report nature that circumvents a great deal of measurements and collection of more objective data.

## Ethics Statement

The Institutional Review Board of Qatar University approved the research design and tools prior to conducting the study (approval no. QU-IRB 744-E/17). An informed consent form was attached to the questionnaire to explain the requirements of the study and its purpose to the participants, and to obtain their agreement to participate voluntarily. The confidentiality of all participants' information was managed ethically and professionally by researchers during collecting, analyzing, and storing of the data.

## Author Contributions

FH conceived and designed the initial experiments. FH, RD, TE-S, HA-M, and ZA-R finalized the design of the study and performed the experiments. FH, RD, TE-S, HA-M, ZA-R, and OA analyzed the data. FH and OA coordinated the writing of this manuscript with the contribution of RD, TE-S, HA-M, and ZA-R.

### Conflict of Interest Statement

The authors declare that the research was conducted in the absence of any commercial or financial relationships that could be construed as a potential conflict of interest.
