# Supplementary material for: The Relationship Between Sedentary Behavior, Back Pain, and Psychosocial Correlates Among University Employees
Source: Front Public Health. 2019 Apr 9;7:80. doi: 10.3389/fpubh.2019.00080 (PMC6465323; doi:10.3389/fpubh.2019.00080)
Supplement: Supplementary file 1 [file Data_Sheet_1.docx]

Appendix

Table S1: List of items extracted from the three questionnaire. (Demographic characteristics are not included)

| No. | Item | Type |
| --- | --- | --- |
| 1 | How much time do you usually spend sitting or reclining on a typical day? Work/non work related. | Continuous |
| 2 | During the last 7 days, on how many days did you walk for at least 10 minutes at a time as part of your work? | Continuous |
| 3 | How much time did you usually spend on one of those days walking as part of your work? | Continuous |
| 4 | During the last 7 days, on how many days did you travel in a motor vehicle such as a bus or car? | Continuous |
| 5 | How much time did you usually spend on one of those days traveling in a bus or car? | Continuous |
| 6 | Once again, think about only those physical activities that you did for at least 10 minutes at a time. During the last 7 days, on how many days did you do moderate activities like (carrying light loads, doing housework, cooking and/or ) inside your home? | Continuous |
| 7 | How much time did you usually spend on one of those days doing moderate physical activities inside your home? | Continuous |
| 8 | How much time do you usually spend sitting or reclining on a typical day? General. | Continuous |
| 9 | Do you do any vigorous-intensity sports, fitness or recreational (leisure) activities that cause large increases in breathing or heart rate like [running or football,] for at least 10 minutes continuously? | Categorical |
| 10 | In a typical week, on how many days do you do vigorous-intensity sports, fitness or recreational (leisure) activities? | Continuous |
| 11 | How much time do you spend doing vigorous-intensity sports, fitness or recreational activities on a typical day? | Continuous |
| 12 | Do you do any moderate-intensity sports, fitness or recreational (leisure) activities that causes a small increase in breathing or heart rate such as brisk walking,(cycling, swimming, volleyball)for at least 10 minutes continuously? | Categorical |
| 13 | In a typical week, on how many days do you do moderate-intensity sports, fitness or recreational (leisure) activities? | Continuous |
| 14 | How much time do you spend doing moderate-intensity sports, fitness or recreational (leisure) activities on a typical day? | Continuous |
| 15 | Have you experienced any back pain recently? | Categorical |
| 16 | How long have you had your back pain problem? | Categorical |
| 17 | How many days of work have you missed because of back pain during the last 18 months? | Categorical |
| 18 | How would you rate the pain that you have had during the past 3 months? | Likert scale 1-5 |
| 19 | How often would you say that you have experienced pain episodes, on average, during the past 3 months? | Likert scale 1-5 |
| 20 | Based on all the things you do to cope, or deal with your pain, on an average day, how much are you able to decrease it? | Likert scale 1-5 |
| 21 | How tense or anxious have you felt because of your back pain in the past 3 months? | Likert scale 1-5 |
| 22 | How much have you been bothered by depressed feeling in the past 3 months? | Likert scale 1-5 |
| 23 | In your view, how large is the risk that your current pain may become continual? | Likert scale 1-5 |
| 24 | An increase in pain is an indication that I should stop what I am doing until the pain decreases | Likert scale 1-5 |
